# A performance validation of six commercial wrist-worn wearable sleep-tracking devices for sleep stage scoring compared to polysomnography

**DOI:** 10.1093/sleepadvances/zpaf021

**Published:** 2025-03-22

**Authors:** An-Marie Schyvens, Brent Peters, Nina Catharina Van Oost, Jean-Marie Aerts, Federica Masci, An Neven, Hélène Dirix, Geert Wets, Veerle Ross, Johan Verbraecken

**Affiliations:** Laboratory of Experimental Medicine and Pediatrics, University of Antwerp, Wilrijk, Belgium; Multidisciplinary Sleep Disorders Centre, Antwerp University Hospital, Edegem, Belgium; UHasselt – Hasselt University, Transportation Research Institute (IMOB), Hasselt, Belgium; Department of Biosystems, KU Leuven, Leuven, Belgium; Department of Biosystems, KU Leuven, Leuven, Belgium; Department of Biosystems, KU Leuven, Leuven, Belgium; UHasselt – Hasselt University, Transportation Research Institute (IMOB), Hasselt, Belgium; UHasselt – Hasselt University, Transportation Research Institute (IMOB), Hasselt, Belgium; UHasselt – Hasselt University, Transportation Research Institute (IMOB), Hasselt, Belgium; UHasselt – Hasselt University, Transportation Research Institute (IMOB), Hasselt, Belgium; Faresa, Evidence-Based Psychological Centre, Hasselt, Belgium; Laboratory of Experimental Medicine and Pediatrics, University of Antwerp, Wilrijk, Belgium; Multidisciplinary Sleep Disorders Centre, Antwerp University Hospital, Edegem, Belgium

**Keywords:** sleep, wearable device, validation, performance, polysomnography, assessment of sleep

## Abstract

**Study Objectives:**

The aim of this study is to assess the performance of six different consumer wearable sleep-tracking devices, namely the Fitbit Charge 5, Fitbit Sense, Withings Scanwatch, Garmin Vivosmart 4, Whoop 4.0, and the Apple Watch Series 8, for detecting sleep parameters compared to the gold standard, polysomnography (PSG).

**Methods:**

Sixty-two adults (52 males and 10 females, mean age ± *SD* = 46.0 ± 12.6 years) spent a single night in the sleep laboratory with PSG while simultaneously using two to four wearable devices.

**Results:**

The results indicate that most wearables displayed significant differences with PSG for total sleep time, sleep efficiency, wake after sleep onset, and light sleep (LS). Nevertheless, all wearables demonstrated a higher percentage of correctly identified epochs for deep sleep and rapid eye movement sleep compared to wake (W) and LS. All devices detected >90% of sleep epochs (ie, sensitivity), but showed lower specificity (29.39%–52.15%). The Cohen’s kappa coefficients of the wearable devices ranged from 0.21 to 0.53, indicating fair to moderate agreement with PSG.

**Conclusions:**

Our results indicate that all devices can benefit from further improvement for multistate categorization. However, the devices with higher Cohen’s kappa coefficients, such as the Fitbit Sense (κ = 0.42), Fitbit Charge 5 (κ = 0.41), and Apple Watch Series 8 (κ = 0.53), could be effectively used to track prolonged and significant changes in sleep architecture.

**Statement of Significance**

This study provides an updated evaluation of the performance of six popular consumer wearable sleep-tracking devices against the gold standard of polysomnography for sleep assessment. This research validates the performance of the sleep-tracking wearables against the gold standard reference for measuring sleep and benefits from a relatively large sample size of 62 participants. The novelty lies in its comprehensive evaluation of multiple devices, offering clinicians and researchers a clear understanding of their accuracy and limitations.

Sleep is increasingly recognized as important for health and well-being [1]. In addition to this growing awareness, wearable devices for sleep tracking have gained immense popularity over the past few years [2]. Wearables are devices designed to measure various physiological parameters while worn on the body. Sleep-tracking devices are a growing trend in wearable health technology by providing users with detailed data on sleep architecture and hypnograms through their associated apps, offering insights into sleep stages and patterns [2, 3].

Polysomnography (PSG) is the gold-standard method for objectively assessing sleep. During PSG, signals of brain activity, eye movements, and muscle tone, as well as audio and video, are recorded, enabling classification of sleep stages in detail [4]. However, PSG may not be ideal for monitoring sleep in particular settings, as it is expensive, labor-intensive, and time-consuming; requires various equipment and technical and medical expertise; and is impractical for long-term use or in-home environment settings [5, 6]. In addition, applying and removing the sensors, organizing the patient administration, and thoroughly analyzing the data that PSG adds is quite labor-intensive for sleep experts. Besides applying and removing the sensors and possibly completing questionnaires and other administrative tasks, PSG requires an overnight stay in a sleep clinic or laboratory, which makes PSG time-consuming for both the sleep technicians and patients. Due to the inherent limitations of PSG, sleep-tracking wearables using an accelerometer and photoplethysmography (PPG) are being explored as a feasible alternative, largely due to their lower cost, convenience, and ability to measure sleep in both clinical and personal settings.

Accelerometers and PPG sensors monitor different movement and physiological patterns throughout the night. Accelerometers are small electromechanical devices that measure acceleration along multiple axes (usually three axes: *x*, *y*, and *z*) to detect positional changes, turning over, or significant body movements during the night [7]. Due to the body movement variations specific to each sleep stage, accelerometers can provide information about wakefulness and general sleep stages. However, they may tend to overestimate sleep due to poorly distinguishing between sleep and restful supine wake periods (eg, lying down while reading or watching television), or they could underestimate sleep due to potential body movements during sleep being categorized as awakenings [7–10]. By combining an accelerometer with a PPG sensor in wearables, a more comprehensive and accurate assessment of sleep could be provided. PPG sensors are a noninvasive technology using a light source and a photodetector at the surface of the skin to measure the volumetric variations of blood circulation and thus can be used to monitor heart rate, heart rate variability, blood flow, and blood oxygen levels [11, 12]. Due to the specific cardiovascular features of each sleep stage, PPG can provide more information about the sleep stages in addition to the accelerometer [3, 12]. The benefits of these sensors used in wearables include their low cost, noninvasive nature, and ability to provide continuous monitoring and real-time data. However, the readings of PPG can be affected by motion artifacts, skin pigmentation, or tissue thickness. In addition, they could be susceptible to environmental factors such as ambient light and temperature [13–15].

Although many researchers and clinicians have doubts about their accuracy in monitoring sleep, wearable sleep-tracking devices are widely used and becoming more technologically advanced, creating strong interest from researchers and clinicians for their possible use as alternatives to PSG.

While several studies have assessed the performance of consumer sleep-tracking devices, continuous advancements in wearable technology involve the validation of newer models against PSG, the gold standard for sleep measurement [16–19]. In particular, devices such as the Fitbit Charge 5, Withings Scanwatch, Garmin Vivosmart 4, and WHOOP 4.0 have received limited independent validation in peer-reviewed studies utilizing PSG [3, 20–25]. Many previous investigations have focused on earlier models of these wearables or have relied on alternative reference methods [9, 22, 23].

For instance, Mouritzen et al. [20] conducted a validation study comparing the Garmin Vivosmart 4, against PSG, but only had a sample size of 18 participants. Stone et al. [21] also conducted a validation study of the Garmin Vivosmart 4 but used an ambulatory EEG monitor as a reference tool and had a sample size of only five participants. No validation studies were found for the Fitbit Charge 5. However, few studies were found that validated the Fitbit Charge 4 against PSG, but only with small sample sizes ranging from 2 to 37 participants [9, 22, 23].

Furthermore, the algorithms used by the wearable manufacturers lack transparency, hindering the understanding of their methodological strengths and limitations. To ensure these devices can be confidently adopted in clinical settings or for long-term monitoring, performance validation of the newer wearable models is still needed.

Therefore, the aim of this laboratory-based study was to provide an updated evaluation of the performance of six wearable sleep-tracking devices—Fitbit Charge 5, Fitbit Sense, Withings Scanwatch, Garmin Vivosmart 4, Whoop 4.0, and Apple Watch Series 8—for assessing sleep. The wearables included in this study were chosen based on the following criteria: recent generation, good ease of use (affordable, unobtrusive, and sufficient battery life), and assessment of variables that could also be used for monitoring sleep, stress, fatigue, and sleepiness namely, heart rate, heart rate variability, stress indicator, and activity. To examine the performance, sleep measures derived from the wearable devices were compared to gold-standard measures derived from PSG [2].

## Methods

All procedures contained herein were approved by the Ethics Committee of Antwerp University Hospital on April 3, 2023 (reference number 3126). Written consent was obtained from each participant prior to engagement.

### Participants

Participants aged 18 years or older were selected irrespective of gender. No specific profile was selected, that is, both patients with suspected sleep apnea as well as healthy participants were selected for this study. Sixty-two adults participated in this performance study (52 males and 10 females; mean age ± *SD* = 46.0 ± 12.6 years; mean body mass index [BMI] ± *SD* = 28.8 ± 5.6 kg/m^2^) with measurements of sleep parameters from one night of simultaneous PSG and wearable application.

The sample size for this study (*n* = 62) was determined based on a review of previous research on wearable performance for sleep measurements. Prior studies in this field have utilized smaller sample sizes of 2–12 participants [22–24]. While a few studies with moderately larger sample sizes (37, 53, and 35 participants) were identified, we aimed to exceed these numbers to achieve a more robust and representative dataset [3, 9, 26]. By selecting a sample size of 62, we ensured a relatively large cohort to provide a robust dataset while maintaining a feasible study design within the constraints of in-lab PSG monitoring.

### Procedure

PSG was performed overnight while participants were using two to four different sleep-tracking wearables. All PSGs were performed in the Multidisciplinary Sleep Disorders Center of the Antwerp University Hospital from April 2023 until August 2024. Participants arrived at the sleep center between 1:00 pm and 4:00 pm and were immediately fitted with the PSG equipment. This allowed them sufficient time to acclimatize to both the monitoring apparatus and the sleep laboratory environment. Participants were instructed to follow their usual bedtime routine and went to sleep at their habitual bedtime.

#### Wearables.

Six wrist-worn wearables containing an accelerometer and PPG were tested on the assessment of sleep, that is, Fitbit Charge 5, Fitbit Sense, Withings Scanwatch, Garmin Vivosmart 4, Whoop 4.0, and Apple Watch Series 8. Each of the wearable devices applies their own separate set of proprietary algorithms to these data to assess various sleep metrics.

While data for sleep stages were measured directly by the wearable, wake after sleep onset (WASO) and sleep onset latency (SOL) were extracted directly from the analysis; total sleep time (TST) was calculated by subtracting the amount of WASO from the sleep period time (SPT). Sleep efficiency (SE) was calculated by dividing the TST by the SPT.

Each participant wore two to four wearable devices during the study, with no more than two devices worn per arm. Wearables were worn on both the left and right arm simultaneously to balance placement across participants. The specific number of devices worn depended on the participant’s comfort. Device placement was determined in set positions of which the order was rotated following a predetermined schedule to ensure that no systematic bias was introduced by the position of a particular device.

#### Polysomnography.

Participants were fitted with sleep monitoring equipment for the PSG measurements by experienced sleep technicians. The montage included four channels of electroencephalography (C4-M1, C3-M2, F4-M1, O2-M1) to assess brain activity, two electro-oculograms (left E1-M2 and right E2-M2) to assess eye movements, and electromyograms of the legs and chin to assess muscle tone. As part of the PSG, piezo belts and airflow sensors such as a thermistor and a nasal cannula were used to determine respiration. Lastly, video recordings and a microphone were used to verify body position, breathing and snoring sounds, and talking [27]. Sleep stages were scored according to American Academy of Sleep Medicine (AASM) criteria by a single technician to avoid interrater differences. Each 30-second epoch of time in bed was manually reviewed and scored as wake, stage 1, 2, or 3 non-rapid eye movement (REM) sleep (N1, N2, and N3) or REM sleep. The scored PSG records were subsequently used to determine the amount of time spent in any stage of sleep (total sleep time) and the amount of wake, N1, N2, N3 and REM.

### Data analysis

The wearable sleep data were exported or viewed from the associated wearable apps using the default settings. Descriptive statistics were calculated using Microsoft Excel (2024).

#### Epoch-by-epoch analysis.

An epoch-by-epoch analysis involved breaking down the continuous stream of sleep data of PSG into discrete time intervals called *epochs* [28]. Afterwards, each epoch was compared individually to the corresponding epoch generated by the wearables. To compare the wearable data files with the corresponding PSG data files, their resolutions were matched. As sleep staging is always assessed by 30-second epochs, wearable epochs were converted to match those of the PSG recordings. Thus, a 1-minute wearable epoch, for example, staged as light sleep (LS), was divided into two smaller epochs of 30 seconds, both staged as LS. This was done in accordance with previously performed comparative studies [3, 16]. Wearable and PSG data were compared during the lights-out period (8 hours) of the PSG registration.

Comparing wearable performance with PSG was performed for both two-state categorization and four-state categorization. The two-state categorization allows the determination of (1) the sensitivity for sleep, that is, the percentage of epochs the wearable correctly scored as sleep compared to PSG, and (2) the specificity for wake, that is, the percentage of epochs the wearable correctly scored as wake compared to PSG.

For four-state categorization, data are classified as one of 12–16 types of sleep stages, based on the wearable’s and PSG’s scoring (Table 1).

**Table 1.** Error matrices for classification of sleep stages comparing polysomnography and wearable devices

Wearable					
PSG		W	LS	DS	REM sleep
Wake	True W	False LS	False DS	False REM
N1 + N2	False W	True LS	False DS	False REM
N3	False W	False LS	True DS	False REM
REM	False W	False LS	False DS	True REM

Abbreviations: DS, deep sleep; LS, light sleep; PSG, polysomnography; REM, rapid eye movement; W, wake.

Wearables’ LS was compared to PSG N1 + N2 sleep, wearables’ deep sleep (DS) to PSG N3 sleep, and wearables’ REM sleep to PSG REM sleep. In case of the Withings Scanwatch data, DS was compared to PSG N3 + REM as this device combines both sleep stages.

Four-state categorization was used to determine all other variables, along with their mean differences (bias), lower and upper limits of agreement (LoA), and whether the differences were significant [3]. Statistical significance was set at a *p*-value of <0.05.

While data for sleep stages, WASO, and SOL were extracted directly from the epoch-by-epoch analysis, TST was calculated by subtracting the amount of WASO from the SPT. SE was calculated by dividing the TST by the SPT.

#### Cohen’s kappa coefficient.

In the case of wearable performance studies for sleep, Cohen’s kappa values are often used to assess the agreement between the wearable device’s sleep detection algorithm and a reference standard, such as PSG. In addition to the observed agreement between the devices, Cohen’s kappa values also take into account the possibility that the agreement comes by chance. The interpretation of Cohen’s kappa values is often categorized as follows: values ≤0 indicate no agreement, 0.01–0.20 indicate none to slight agreement, 0.21–0.40 indicate fair agreement, 0.41–0.60 indicate moderate agreement, 0.61–0.80 indicate substantial agreement, and 0.81–1.00 indicate almost perfect agreement [29].

#### Bland–Altman plots.

The Bland–Altman analysis was employed to assess the agreement between the continuous metrics measured by the wearable device, compared to PSG. This method allows for the identification of any mean difference (bias) and the estimation of the 95% LoA. To compare the data of TST, SE, SOL, WASO, wake (W), LS, DS, and REM from the PSGs with those of the wearables, a Bland–Altman plot was used. These plots highlight both systematic bias and variability between methods, offering valuable insights into whether wearables consistently overestimate or underestimate specific parameters. The 95% LoA were calculated as the mean difference between the wearable and PSG measurements ± 1.96 times the standard deviation of the differences. This method allows to evaluate bias between mean differences of two different methods, and to estimate an interval in which 95% of the differences of the other method, compared to the first one, fall. This results in a scatter plot where the y-axis displays the difference between two paired measurements, while the x-axis displays the average of these two measures [3, 30, 31].

Additionally, the mean absolute error (MAE) and mean absolute percentage error (MAPE) were calculated. MAE provides a direct measure of the average magnitude of error, while MAPE contextualizes this error as a percentage of the observed values, allowing for a proportional understanding of the device’s performance [32].

## Results

### Missing data procedures

**Figure 1.**
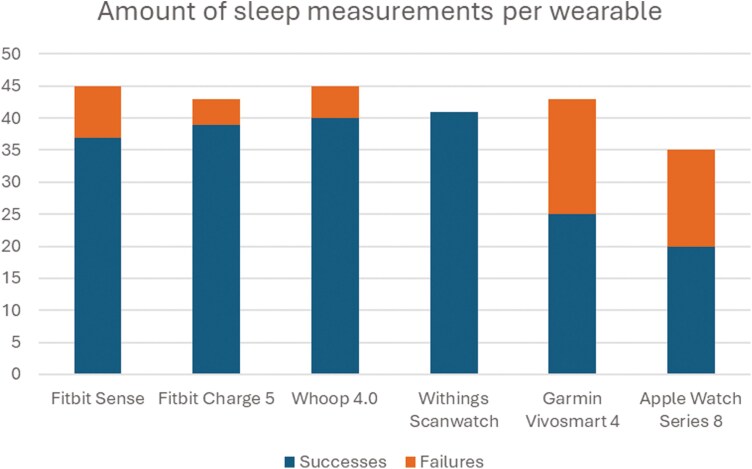
Amount of sleep measurements per wearable. Proportion of successfully captured vs. failed sleep data from wearable devices as a consequence of missing sleep data or partial sleep data loss.

With each lab visit, the research staff carefully synced and charged all the wearable devices in order to capture sleep data on the devices. Despite these efforts, missing sleep data or partial data loss occurred more often than anticipated. Specifically, for the Garmin Vivosmart 4, data were available for 25 participants, while 18 participants had missing data. For the Apple Watch Series 8, data were available for 20 participants, with 15 missing datasets or partial data loss. In addition, no device malfunction or other concerns were observed that may be driving these data losses (Figure 1, Table 2).

**Table 2.** Amount of successfully captured vs. failed sleep data from wearable devices as a consequence of missing sleep data or partial sleep data loss

	Successes	Failures
Fitbit Sense	37	8
Fitbit Charge 5	39	4
Whoop 4.0	40	5
Withings Scanwatch	41	0
Garmin Vivosmart 4	25	18
Apple Watch Series 8	20	15

### Sleep parameters

**Figure 2.**
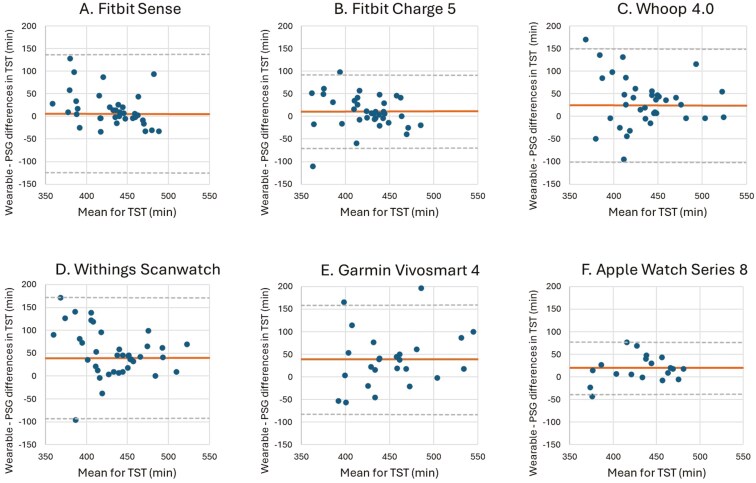
Bland–Altman plots for device-derived and polysomnography (PSG)-derived measures of total sleep time (TST). Bland–Altman plots showing the difference between the (A) Fitbit Sense, (B) Fitbit Charge 5, (C) Whoop 4.0, (D) Withings Scanwatch, (E) Garmin Vivosmart 4 and (F) Apple Watch Series 8 and PSG plotted against the mean of both metrics for TST—positive values indicate devices overestimate relative to PSG, whereas negative values indicate devices underestimate relative to PSG. The plots depict the mean bias (solid line) and upper and lower limits of agreement (2 *SD*s from bias; dashed lines) for minutes of TST for the devices compared with PSG.

**Figure 3.**
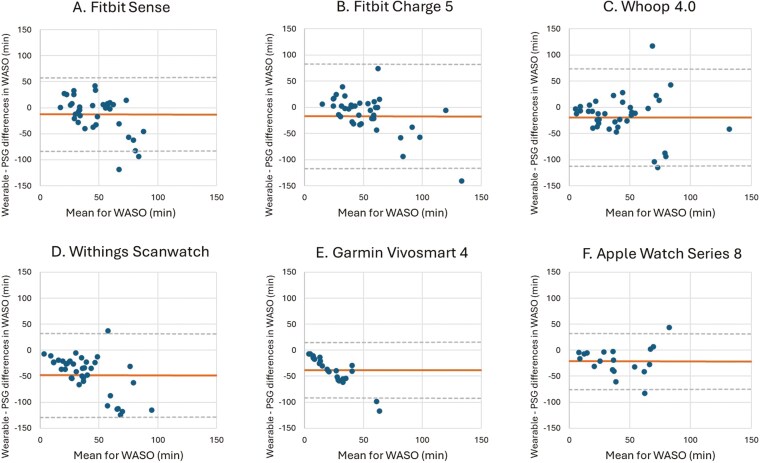
Bland–Altman plots for device-derived and polysomnography (PSG)-derived measures of wake after sleep onset (WASO). Bland–Altman plots showing the difference between the (A) Fitbit Sense, (B) Fitbit Charge 5, (C) Whoop 4.0, (D) Withings Scanwatch, (E) Garmin Vivosmart 4 and (F) Apple Watch Series 8 and PSG plotted against the mean of both metrics for WASO—positive values indicate devices overestimate relative to PSG, and negative values indicate devices underestimate relative to PSG. The plots depict the mean bias (solid line) and upper and lower limits of agreement (2 *SD*s from bias; dashed lines) for minutes of WASO for the devices compared with PSG.

**Figure 4.**
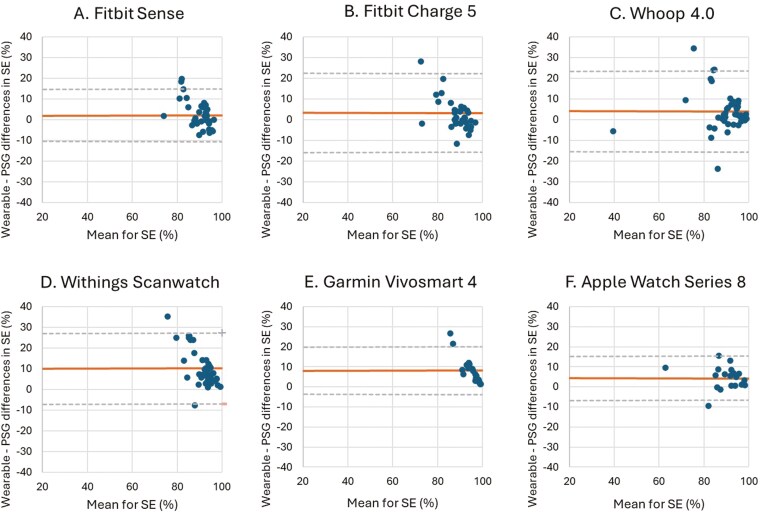
Bland–Altman plots for device-derived and polysomnography (PSG)-derived measures of sleep efficiency (SE). Bland–Altman plots showing the difference between the (A) Fitbit Sense, (B) Fitbit Charge 5, (C) Whoop 4.0, (D) Withings Scanwatch, (E) Garmin Vivosmart 4 and (F) Apple Watch Series 8 and PSG plotted against the mean of both metrics for SE—positive values indicate devices overestimate relative to PSG, and negative values indicate devices underestimate relative to PSG. The plots depict the mean bias (solid line) and upper and lower limits of agreement (2 *SD*s from bias; dashed lines) for minutes of SE for the devices compared with PSG.

**Figure 5.**
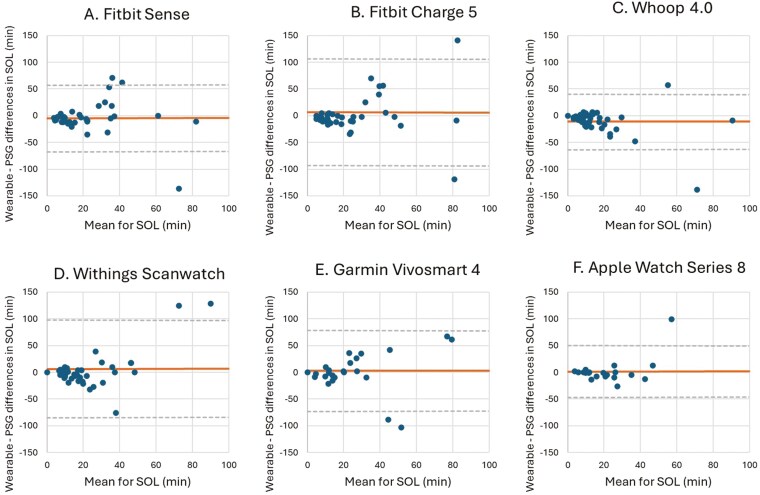
Bland–Altman plots for device-derived and polysomnography (PSG)-derived measures of sleep onset latency (SOL). Bland–Altman plots showing the difference between the (A) Fitbit Sense, (B) Fitbit Charge 5, (C) Whoop 4.0, (D) Withings Scanwatch, (E) Garmin Vivosmart 4 and (F) Apple Watch Series 8 and PSG plotted against the mean of both metrics for SOL—positive values indicate devices overestimate relative to PSG, and negative values indicate devices underestimate relative to PSG. The plots depict the mean bias (solid line) and upper and lower limits of agreement (2 *SD*s from bias; dashed lines) for minutes of SOL for the devices compared with PSG.

The results for the different sleep parameter measures (TST, WASO, SE, and SOL) are shown in Tables 3–6. Corresponding Bland–Altman plots are shown in Figures 2–5.

**Table 3.** Total sleep time (TST) (min) as assessed by polysomnography and wearable devices displayed as mean ± *SD* with associated biases and limits of agreement

Wearable	*n*	PSG, mean ± *SD*	Wearable, mean ± *SD*	Bias	*p*-Value	MAE	MAPE	Upper LoA	Lower LoA
Fitbit Sense	37	421.04 ± 46.56	427.35 ± 75.22	6.31	0.285	35.23	42.08	137.32	−124.70
Fitbit Charge 5	39	408.37 ± 56.07	419.49 ± 51.14	11.12	0.051	31.24	7.81	92.50	−70.27
Whoop 4.0	40	409.95 ± 61.58	434.41 ± 72.64	24.46	**0.010**	50.31	12.61	150.01	−101.09
Withings Scanwatch	41	406.83 ± 52.33	446.70 ± 59.74	39.87	**0.000**	60.94	14.58	172.31	−92.58
Garmin Vivosmart 4	25	432.42 ± 47.64	470.86 ± 59.00	38.44	**0.002**	54.36	11.24	159.51	−82.63
Apple Watch Series 8	20	415.63 ± 46.20	435.23 ± 46.59	19.60	**0.004**	27.75	6.50	77.55	−38.35

Significant *p*-values (*p* < 0.05) are indicated by a bold font. Abbreviations: LoA, limits of agreement; MAE, mean absolute error; MAPE, mean absolute percentage error; PSG, polysomnography.

**Table 4.** Wake after sleep onset (WASO) (min) as assessed by polysomnography and wearable devices displayed as mean ± *SD* with associated biases and limits of agreement

Wearable	*n*	PSG, mean ± *SD*	Wearable, mean ± *SD*	Bias	*p*-Value	MAE	MAPE	Upper LoA	Lower LoA
Fitbit Sense	37	53.92 ± 33.37	41.54 ± 17.49	−12.38	**0.022**	26.19	102.94	58.31	−83.06
Fitbit Charge 5	39	63.62 ± 52.44	46.76 ± 20.43	−16.86	**0.023**	29.91	70.31	83.11	−116.83
Whoop 4.0	40	60.74 ± 55.16	41.59 ± 52.02	−19.15	**0.007**	34.18	153.29	73.73	−112.03
Withings Scanwatch	41	64.43 ± 41.62	16.49 ± 17.37	−47.94	**0.000**	49.74	489.65	32.51	−128.39
Garmin Vivosmart 4	25	42.36 ± 29.36	4.02 ± 6.58	−38.34	**0.000**	38.34	1216.61	15.11	−91.79
Apple Watch Series 8	20	58.00 ± 42.65	36.78 ± 39.18	−21.23	**0.001**	26.48	229.12	32.31	−74.76

Significant *p*-values (*p* < 0.05) are indicated by a bold font. Abbreviations: LoA, limits of agreement; MAE, mean absolute error; MAPE, mean absolute percentage error; PSG, polysomnography.

**Table 5.** Sleep efficiency (SE) (%) as assessed by polysomnography and wearable devices displayed as mean ± *SD* with associated biases and limits of agreement

Wearable	*n*	PSG, mean ± *SD*	Wearable, mean ± *SD*	Bias	*p*-Value	MAE	MAPE	Upper LoA	Lower LoA
Fitbit Sense	37	88.59 ± 7.20	90.79 ± 4.16	2.20	**0.024**	4.83	5.31	14.93	−10.54
Fitbit Charge 5	39	86.53 ± 11.0	89.92 ± 4.40	3.39	**0.015**	5.97	6.76	22.42	−15.64
Whoop 4.0	40	87.04 ± 11.64	91.14 ± 10.96	4.10	**0.006**	7.14	8.07	23.63	−15.42
Withings Scanwatch	41	86.28 ± 8.81	96.47 ± 3.62	10.19	**0.000**	10.57	10.95	27.36	−6.98
Garmin Vivosmart 4	25	90.95 ± 6.46	99.12 ± 1.44	8.17	**0.000**	8.17	8.26	19.97	−3.63
Apple Watch Series 8	20	87.80 ± 8.77	92.25 ± 8.08	4.44	**0.001**	5.53	6.14	15.40	−6.51

Significant *p*-values (*p* < 0.05) are indicated by a bold font. Abbreviations: LoA, limits of agreement; MAE, mean absolute error; MAPE, mean absolute percentage error; PSG, polysomnography.

**Table 6.** Sleep onset latency (SOL) (min) as assessed by polysomnography and wearable devices displayed as mean ± *SD* with associated biases and limits of agreement

Wearable	*n*	PSG, mean ± *SD*	Wearable, mean ± *SD*	Bias	*p*-Value	MAE	MAPE	Upper LoA	Lower LoA
Fitbit Sense	37	27.30 ± 31.63	22.95 ± 26.09	−4.35	0.206	18.46	250.90	58.02	−66.72
Fitbit Charge 5	39	24.31 ± 25.38	30.87 ± 44.61	6.56	0.212	25.59	133.31	106.11	−92.99
Whoop 4.0	40	22.79 ± 25.83	11.84 ± 18.10	−10.95	**0.006**	15.30	451.96	40.57	−62.47
Withings Scanwatch	41	24.18 ± 23.77	30.77 ± 46.38	6.59	0.185	22.71	107.96	97.45	−84.28
Garmin Vivosmart 4	25	29.44 ± 33.69	32.28 ± 44.24	2.84	0.357	24.88	159.43	77.85	−72.17
Apple Watch Series 8	20	21.00 ± 13.17	22.88 ± 23.26	1.88	0.368	11.23	46.52	50.10	−46.35

Significant *p*-values (*p* < 0.05) are indicated by a bold font. Abbreviations: LoA, limits of agreement; MAE, mean absolute error; MAPE, mean absolute percentage error; PSG, polysomnography.

The mean difference (bias) between the wearable devices, except Fitbit Charge 5, and PSG indicated that, on average, the wearables overestimated TST by 6.31–39.87 minutes compared to PSG. The Fitbit Charge 5 displayed a mean difference of −5.74 minutes for TST compared to PSG, indicating an underestimation of the TST by 5.74 minutes compared to PSG (Table 3). The bias for TST was not significant when measured by the Fitbit Charge 5 (*p* = 0.051) and the Fitbit Sense (*p* = 0.285). The wide LoA indicate that there is a considerable degree of variability in the differences between the wearable devices and PSG (Figure 2). This suggests that while the wearable devices such as the Fitbit Sense and the Fitbit Charge 5 generally agree with PSG, the wider LOA indicates that for some individuals, sleep parameters may be considerably different from PSG. For individuals with lower TST, the tendency to overestimate may arise from the wearable’s inability to accurately detect short wake episodes interspersed within sleep periods, leading to an inflation of the calculated sleep time. Conversely, for individuals with higher TST, underestimation may occur due to difficulties in distinguishing between periods of restful wakefulness and LS, especially during prolonged sleep durations, which can result in misclassification and a reduction in the reported TST [33]. The Apple Watch Series 8 showed the most narrow LoA (−38.35 to 77.55 minutes) indicating that the Apple Watch Series 8 showed the least amount of variability in the differences with PSG. MAE values for TST ranged from 27.75 minutes (Apple Watch Series 8) to 60.94 minutes (Withings Scanwatch). Lower MAE values, such as those observed with the Apple Watch and Fitbit Charge 5, suggest these devices are better at estimating total sleep duration compared to others like the Garmin Vivosmart 4 or Withings Scanwatch. The MAPE values further highlight the Apple Watch’s superior relative accuracy (6.5%), while devices with higher MAPEs may struggle with consistent performance across different sleep scenarios (Figure 2, Table 3).

Additionally, the Bland–Altman plots for TST showed no apparent pattern in the distribution of errors, suggesting that the magnitude of the discrepancies between wearable devices and PSG does not systematically vary across different TST values (Figure 2).

The mean differences between the wearable devices and PSG for WASO indicated that, on average, all the wearable devices significantly underestimated WASO by 12.38–47.94 minutes compared to PSG. The wide LoA, especially for the Fitbit Charge 5 (−116.83 to 83.11 minutes) and the Whoop 4.0 (−112.03 to 73.73 minutes) indicate that there is a considerable degree of variability in the differences between the wearable devices and PSG. The Apple Watch Series 8 (−74.76 to 32.31 minutes), the Garmin Vivosmart (−91.79 to 15.11 minutes), the Withings Scanwatch (−128.39 to 32.51 minutes), and the Fitbit Sense (−83.06 to 58.31 minutes) showed the least wide LoA indicating that the degree of variability in the differences is smaller between these wearable devices with PSG. The MAE and MAPE for WASO revealed striking differences between devices. While devices like the Fitbit Sense and Apple Watch Series 8 had lower MAE values (26–27 minutes), others, such as the Garmin Vivosmart 4, showed extremely high MAPE values (eg, 1216.61%), indicating significant challenges in detecting wake periods after initial sleep onset (Figure 3, Table 4).

The mean differences between the wearable devices and PSG for SE indicated that, on average, all the wearable devices significantly overestimated SE by 2.20 (*p* = 0.024) to 10.19% (*p* < 0.001) compared to PSG. The LoA showed a similar range between the different wearable devices indicating that the degree of variability in the differences between the wearable devices and PSG is similar for all of the assessed devices. The relatively low MAEs (4.83–10.57%) and MAPEs (5.31–10.95%) for SE across devices indicate that most wearables provide reasonably accurate estimates of overall sleep efficiency (Figure 4, Table 5).

For measuring SOL, no significant mean differences were shown by the Fitbit Sense (−4.35 minutes, *p* = 0.206), Fitbit Charge 5 (6.56 minutes, *p* = 0.212), Withings Scanwatch (6.59 minutes, *p* = 0.185), Garmin Vivosmart (2.84 minutes, *p* = 0.357), and Apple Watch Series 8 (1.88 minutes, *p* = 0.368) compared to PSG. Only the Whoop 4.0 showed a significant underestimation (−10.95 minutes, *p* = 0.006) for SOL compared to PSG. The wide LoA indicate that there is a considerable degree of variability in the differences between all the wearable devices and PSG, suggesting that individual measurements differ significantly. In addition, SOL showed some of the largest relative errors, with devices like the Whoop 4.0 and Garmin Vivosmart 4 exhibiting MAPEs of 451.96% and 159.43%, respectively. These errors suggest that wearables face challenges in accurately capturing the transition from wakefulness to sleep (Figure 5, Table 6).

Epoch-by-epoch comparisons indicated quite high sensitivities (91.68%–96.27%). Specificity ranged between 29.39% and 52.15%. The Cohen’s kappa values of the Fitbit Sense (κ = 0.42), Fitbit Charge 5 (κ = 0.41), and the Apple Watch Series 8 (κ = 0.53) indicate a moderate agreement between the device and PSG. The Cohen’s kappa values of the Whoop 4.0 (κ = 0.37), the Withings Scanwatch (κ = 0.22), and the Garmin Vivosmart 4 (κ = 0.21) indicate a fair agreement between the device and PSG (Table 7).

**Table 7.** Kappa, sensitivity, specificity, agreement of wearable devices compared to polysomnography

Wearable	*n*	Kappa, mean ± *SD*	Sensitivity, mean ± *SD* (%)	Specificity, mean ± *SD* (%)
Fitbit Sense	37	0.42 ± 0.14	93.33 ± 3.85	48.80 ± 20.67
Fitbit Charge 5	39	0.41 ± 0.15	91.68 ± 7.28	47.51 ± 17.37
Whoop 4.0	40	0.37 ± 0.16	93.58 ± 9.42	40.13 ± 23.55
Withings Scanwatch	41	0.22 ± 0.15	94.32 ± 10.94	31.09 ± 20.12
Garmin Vivosmart 4	25	0.21 ± 0.13	95.92 ± 6.10	29.39 ± 26.38
Apple Watch Series 8	20	0.53 ± 0.16	96.27 ± 4.57	52.15 ± 21.25

### Sleep stages

**Figure 6.**
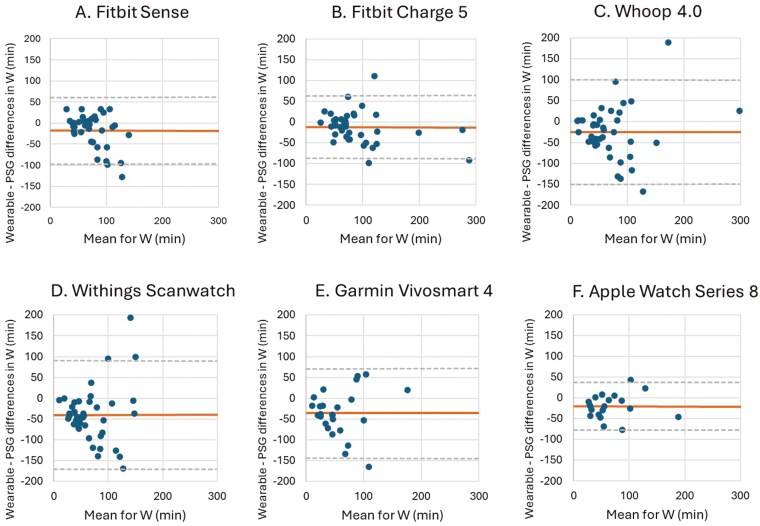
Bland–Altman plots for device-derived and polysomnography (PSG)-derived measures of wake (W). Bland–Altman plots showing the difference between the (A) Fitbit Sense, (B) Fitbit Charge 5, (C) Whoop 4.0, (D) Withings Scanwatch, (E) Garmin Vivosmart 4 and (F) Apple Watch Series 8 and PSG plotted against the mean of both metrics for W—positive values indicate devices overestimate relative to PSG, and negative values indicate devices underestimate relative to PSG. The plots depict the mean bias (solid line) and upper and lower limits of agreement (2 *SD*s from bias; dashed lines) for minutes of W for the devices compared with PSG.

**Figure 7.**
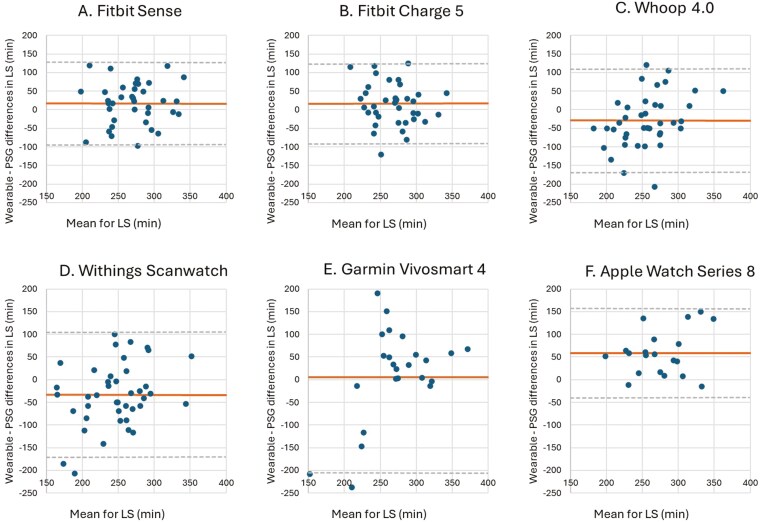
Bland–Altman plots for device-derived and polysomnography (PSG)-derived measures of light sleep (LS). Bland–Altman plots showing the difference between the (A) Fitbit Sense, (B) Fitbit Charge 5, (C) Whoop 4.0, (D) Withings Scanwatch, (E) Garmin Vivosmart 4 and (F) Apple Watch Series 8 and PSG plotted against the mean of both metrics for LS—positive values indicate devices overestimate relative to PSG, and negative values indicate devices underestimate relative to PSG. The plots depict the mean bias (solid line) and upper and lower limits of agreement (2 *SD*s from bias; dashed lines) for minutes of LS for the devices compared with PSG.

**Figure 8.**
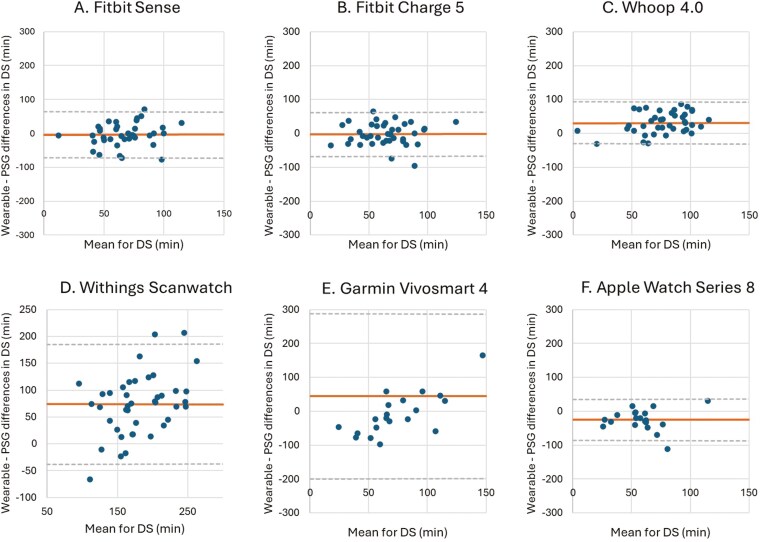
Bland–Altman plots for device-derived and polysomnography (PSG)-derived measures of deep sleep (DS). Bland–Altman plots showing the difference between the (A) Fitbit Sense, (B) Fitbit Charge 5, (C) Whoop 4.0, (D) Withings Scanwatch, (E) Garmin Vivosmart 4 and (F) Apple Watch Series 8 and PSG plotted against the mean of both metrics for DS—positive values indicate devices overestimate relative to PSG, and negative values indicate devices underestimate relative to PSG. The plots depict the mean bias (solid line) and upper and lower limits of agreement (2 *SD*s from bias; dashed lines) for minutes of DS for the devices compared with PSG.

**Figure 9.**
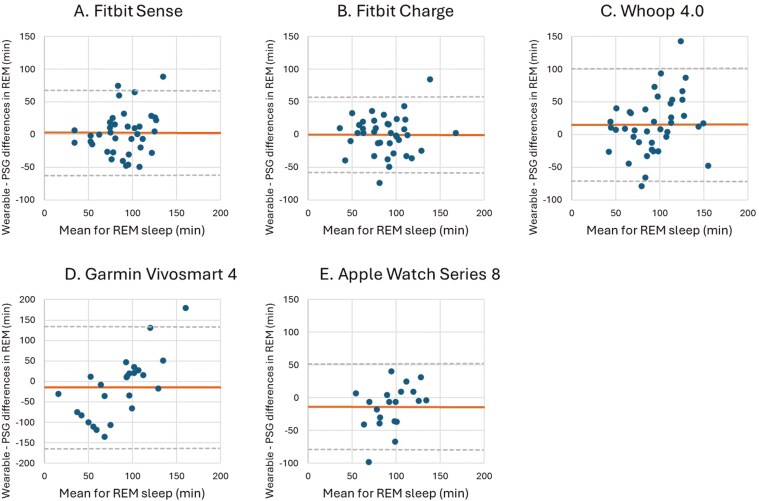
Bland–Altman plots for device-derived and polysomnography (PSG)-derived measures of rapid eye movement (REM) sleep. Bland–Altman plots showing the difference between the (A) Fitbit Sense, (B) Fitbit Charge 5, (C) Whoop 4.0, (D) Garmin Vivosmart 4 and (E) Apple Watch Series 8 and PSG plotted against the mean of both metrics for REM sleep—positive values indicate devices overestimate relative to PSG, and negative values indicate devices underestimate relative to PSG. The plots depict the mean bias (solid line) and upper and lower limits of agreement (2 *SD*s from bias; dashed lines) for minutes of REM sleep for the devices compared with PSG.

The results for the different sleep stage measures (W, LS, DS, and REM sleep) are presented in Tables 8–11. Corresponding Bland–Altman plots are shown in Figures 6–9.

**Table 8.** Wake (W) (min) as assessed by polysomnography and wearable devices displayed as mean ± *SD* with associated biases and limits of agreement

Wearable	*n*	PSG, mean ± *SD*	Wearable, mean ± *SD*	Bias	*p*-Value	MAE	MAPE	Upper LoA	Lower LoA
Fitbit Sense	37	83.66 ± 42.08	66.28 ± 25.90	−17.38	**0.006**	29.62	51.08	61.90	−96.65
Fitbit Charge 5	39	94.78 ± 64.73	83.03 ± 53.45	−11.76	**0.032**	29.53	40.60	63.71	−87.22
Whoop 4.0	40	84.79 ± 57.13	60.29 ± 63.64	−24.50	**0.010**	50.20	215.32	100.69	−146.69
Withings Scanwatch	41	89.04 ± 47.13	49.46 ± 53.26	−39.57	**0.000**	60.65	320.52	91.01	−170.16
Garmin Vivosmart 4	25	75.20 ± 45.04	39.39 ± 49.14	−35.82	**0.002**	51.74	1175.52	71.76	−143.40
Apple Watch Series 8	20	76.38 ± 41.85	56.20 ± 44.23	−20.18	**0.003**	28.33	99.26	37.56	−77.91

Significant *p*-values (*p* < 0.05) are indicated by a bold font. Abbreviations: LoA, limits of agreement; MAE, mean absolute error; MAPE, mean absolute percentage error; PSG, polysomnography.

**Table 9.** Light sleep (LS) (min) as assessed by polysomnography and wearable devices displayed as mean ± *SD* with associated biases and limits of agreement

Wearable	*n*	PSG, mean ± *SD*	Wearable, mean ± *SD*	Bias	*p*-Value	MAE	MAPE	Upper LoA	Lower LoA
Fitbit Sense	37	261.11 ± 44.74	278.88 ± 47.51	17.77	**0.032**	48.58	18.05	128.62	−93.08
Fitbit Charge 5	39	265.67 ± 73.60	282.24 ± 73.31	16.58	**0.034**	45.35	16.76	124.28	−91.12
Whoop 4.0	40	266.65 ± 47.79	238.11 ± 62.30	−28.54	**0.008**	61.71	30.13	110.70	−167.77
Withings Scanwatch	41	262.57 ± 46.73	229.06 ± 63.34	−33.51	**0.002**	61.83	35.57	104.70	−170.17
Garmin Vivosmart 4	25	263.28 ± 48.35	269.08 ± 95.00	5.80	0.395	79.76	61.36	217.17	−205.57
Apple Watch Series 8	20	246.08 ± 41.12	304.83 ± 52.48	58.75	**0.000**	61.40	19.12	156.63	−39.13

Significant *p*-values (*p* < 0.05) are indicated by a bold font. Abbreviations: LoA, limits of agreement; MAE, mean absolute error; MAPE, mean absolute percentage error; PSG, polysomnography.

**Table 10.** Deep sleep (DS) (min) as assessed by polysomnography and wearable devices displayed as mean ± *SD* with associated biases and limits of agreement

Wearable	*n*	PSG, mean ± *SD*	Wearable, mean ± *SD*	Bias	*p*-Value	MAE	MAPE	Upper LoA	Lower LoA
Fitbit Sense	37	68.84 ± 24.62	64.95 ± 29.51	−3.89	0.249	26.16	65.18	63.90	−71.69
Fitbit Charge 5	39	65.51 ± 27.02	63.32 ± 27.68	−2.19	0.339	26.14	51.00	62.04	−66.42
Whoop 4.0	40	60.59 ± 23.29	92.08 ± 32.32	31.49	**0.000**	36.51	53.01	93.08	−30.11
Withings Scanwatch	41	143.88 ± 40.15	217.07 ± 59.12	73.20	**0.000**	78.93	35.69	185.84	−37.11
Garmin Vivosmart 4	25	75.92 ± 22.07	120.36 ± 116.67	44.44	**0.043**	90.72	340.14	288.18	−199.30
Apple Watch Series 8	20	71.00 ± 24.72	45.80 ± 26.16	−25.20	**0.001**	31.20	160.65	36.06	−86.46

Significant *p*-values (*p* < 0.05) are indicated by a bold font. Abbreviations: LoA, limits of agreement; MAE, mean absolute error; MAPE, mean absolute percentage error; PSG, polysomnography.

**Table 11.** Rapid eye movement (REM) sleep (min) as assessed by polysomnography and wearable devices displayed as mean ± *SD* with associated biases and limits of agreement

Wearable	*n*	PSG, mean ± *SD*	Wearable, mean ± *SD*	Bias	*p*-Value	MAE	MAPE	Upper LoA	Lower LoA
Fitbit Sense	37	87.88 ± 27.28	90.74 ± 32.97	2.86	0.302	25.11	28.30	67.97	−62.24
Fitbit Charge 5	39	88.35 ± 29.80	88.10 ± 31.55	−0.24	0.479	21.29	29.69	57.88	−58.23
Whoop 4.0	40	85.81 ± 31.53	101.09 ± 41.12	15.28	**0.017**	35.28	38.69	101.58	−71.03
Garmin Vivosmart 4	25	92.20 ± 27.61	77.78 ± 66.70	−14.42	0.176	59.26	60.16	134.77	−163.61
Apple Watch Series 8	20	101.48 ± 21.55	88.10 ± 32.71	−13.38	**0.045**	26.03	52.58	52.19	−78.94

Significant *p*-values (*p* < 0.05) are indicated by a bold font. Abbreviations: LoA, limits of agreement; MAE, mean absolute error; MAPE, mean absolute percentage error; PSG, polysomnography.

All the wearables displayed a significant underestimation of W compared to PSG (11.76–39.57 minutes). In addition, the MAE and MAPE values vary widely across devices. For example, the Fitbit Sense exhibits moderate MAE (29.62 minutes) and MAPE (51.08%), suggesting a reasonable accuracy for detecting wake durations.

In contrast, the Garmin Vivosmart 4 shows extremely high MAPE (1175.52%), highlighting poor reliability in measuring wake times. Devices like the Apple Watch Series 8 (MAE: 28.33 minutes, MAPE: 99.26%) offer slightly better results than Garmin but are still less accurate compared to the Fitbit models.

The Whoop 4.0, Withings Scanwatch, and Garmin Vivosmart 4 showed wider LoA than the Fitbit Sense, Fitbit Charge 5, and the Apple Watch Series 8, indicating that the degree of variability in the differences is larger for the Whoop 4.0, Scanwatch, and Garmin Vivosmart 4. This suggests that the individual measurements of these devices could differ more than the measurements of the Fitbit Sense, Fitbit Charge 5, and the Apple Watch Series 8 (Figure 6, Table 8).

LS was significantly underestimated by the Whoop 4.0 (28.54 minutes, *p* = 0.008) and Withings Scanwatch (33.51 minutes, *p* = 0.002) and was significantly overestimated by the Fitbit Sense (17.77 minutes, *p* = 0.032), Fitbit Charge 5 (16.58 minutes, *p* = 0.034), and the Apple Watch Series 8 (58.75 minutes, *p* < 0.001). The Garmin Vivosmart 4 showed no significant difference with PSG with an average difference of 5.80 minutes for LS (*p* = 0.395). The MAE and MAPE scores suggest a mixed performance among wearables. The Fitbit Charge 5 (MAE: 45.35 minutes, MAPE: 16.76%) outperforms others, indicating better precision in estimating LS.

The Garmin Vivosmart 4 and Withings Scanwatch have notably higher error (61.36% and 35.57%, respectively), reflecting less consistent outcomes.

The Whoop 4.0 (MAPE: 30.13%) performs reasonably well but still trails behind the Fitbit Charge 5 in LS accuracy (Table 9). The LoA are relatively wide for LS for all the wearable devices, and the differences are evenly distributed around the mean bias, with no apparent pattern. This suggests that the error between the wearable and PSG is consistent across different measurements (Figure 7).

The Fitbit Sense and Fitbit Charge 5 did not display any significant differences for DS and REM sleep compared to PSG. The Whoop 4.0 significantly overestimated both DS (31.49 minutes, *p* < 0.001) and REM sleep (15.28 minutes, *p* = 0.017), while the Apple Watch Series 8 significantly underestimated both DS (25.20 minutes, *p* = 0.001) and REM sleep (13.38 minutes, *p* = 0.045). The Garmin Vivosmart 4 showed a significant overestimation for DS (44.44 minutes, *p* = 0.043), but no significant difference for REM sleep (14.42 minutes, *p* = 0.176) compared to PSG. The MAE and MAPE values for DS reflect significant challenges in wearable performance. While Fitbit devices demonstrate relatively lower MAPE (Sense: 18.05%, Charge 5: 16.76%), the Garmin Vivosmart 4 (MAE: 79.76 minutes, MAPE: 61.36%) struggles considerably in accuracy. The performance for REM sleep detection is inconsistent, with most devices showing high MAPE values. For instance, the Garmin Vivosmart 4 (MAE: 59.26 minutes, MAPE: 60.16%) and Apple Watch Series 8 (MAE: 26.03 minutes, MAPE: 52.58%) suggest limited reliability.

Fitbit devices provide slightly better results, with the Charge 5 exhibiting a MAPE of 29.69%, indicating a moderate level of accuracy (Tables 10 and 11).

The LoA for both DS and REM sleep of the Garmin Vivosmart 4 are notably wide (−199.30 to 288.18 minutes and −163.61 to 134.77 minutes, respectively), indicating that the individual measurements of the Garmin Vivosmart 4 differed significantly compared to PSG. No device malfunction or other concerns were observed that may be driving these results. The LoA for the other wearable devices are narrow compared to the LoA of the Garmin Vivosmart, indicating that for these wearables, the individual measurements differ less than those of the Garmin Vivosmart (Tables 10 and 11, Figures 8 and 9).

The measurements for DS of the Withings Scanwatch (encompassing both N3 sleep and REM sleep) showed a mean difference of 73.20 minutes when compared to the N3 and REM sleep measurements of PSG. This indicates that the Withings Scanwatch significantly overestimates N3 and REM sleep by 73.20 minutes (*p* < 0.001) when compared to PSG. In addition, the wide LoA (−37.11 to 185.84 minutes) indicates that there is a considerable degree of variability in the differences between all the wearable devices and PSG (Table 10, Figure 8).

The Apple Watch Series 8 achieved the highest accuracy in identifying wake epochs, correctly classifying 52.15% of PSG W epochs, followed by the Fitbit Sense (48.80%), Fitbit Charge 5 (47.67%), Whoop 4.0 (40.13%), Withings Scanwatch (31.09%), and Garmin Vivosmart 4 (27.64%). Common errors included misclassification of PSG W epochs as LS, with the Garmin Vivosmart 4 misclassifying 46.35% of W as LS, followed by the Withings Scanwatch (53.89%), Apple Watch Series 8 (42.10%), Fitbit Charge 5 (41.31%), Fitbit Sense (40.46%), and Whoop 4.0 (44.12%).

For LS, the Apple Watch Series 8 showed the highest accuracy, correctly identifying 83.27% of PSG LS epochs. This was followed by the Fitbit Sense (73.30%), Fitbit Charge 5 (72.42%), Whoop 4.0 (61.99%), Garmin Vivosmart 4 (60.33%), and Withings Scanwatch (53.04%). Misclassification of other stages as LS was notable, particularly for N3 and REM sleep, with the Fitbit Sense misclassifying 39.76% of PSG N3 and 30.23% of PSG REM sleep as LS. Similarly, the Fitbit Charge 5 misclassified 37.25% of N3 and 30.48% of REM as LS, with similar trends observed across other devices.

The Whoop 4.0 performed best for identifying DS, correctly classifying 69.63% of PSG N3 epochs. This was followed by the Withings Scanwatch (66.74%), Fitbit Charge 5 (51.50%), Fitbit Sense (50.86%), Apple Watch Series 8 (50.66%), and Garmin Vivosmart 4 (47.46%). Misclassification of PSG N3 as LS occurred frequently, with rates of 47.73% for the Apple Watch Series 8, 46.00% for Garmin Vivosmart 4, and between 22.50% and 41.13% for other devices.

Accuracy in identifying REM sleep epochs was highest for the Apple Watch Series 8, which correctly classified 68.57% of PSG REM epochs. Following this were the Whoop 4.0 (61.99%), Fitbit Sense (61.29%), Fitbit Charge 5 (59.96%), and Garmin Vivosmart 4 (33.10%). Common errors included misclassifying REM as LS, with Garmin Vivosmart 4 and Fitbit Sense misclassifying 41.57% and 30.23% of PSG REM as LS, respectively.

The Withings Scanwatch grouped stages N3 and REM as DS, with 66.74% accuracy for PSG epochs. Common misclassifications included PSG W as LS (53.89%), PSG N1/N2 as DS (41.13%), and PSG N3 or REM sleep as LS (27.69%) (Table 12).

**Table 12.** Error matrices for classification of sleep stages for each device compared to polysomnography (%)

		Wake	Light sleep	Deep sleep	REM
Fitbit Sense					
PSG	Wake	**48.80**	40.46	2.34	8.41
	N1 or N2	7.73	**73.30**	9.33	9.63
	N3	3.94	39.76	**50.86**	5.44
	REM	6.04	30.23	2.44	**61.29**

Fitbit Charge 5					
PSG	Wake	**47.67**	41.31	4.55	6.47
	N1 or N2	9.10	**72.42**	8.40	10.08
	N3	9.20	37.25	**51.50**	2.05
	REM	6.17	30.48	3.39	**59.96**

Whoop 4.0					
PSG	Wake	**40.13**	44.12	6.17	9.58
	N1 or N2	7.04	**61.99**	14.82	16.14
	N3	3.12	22.50	**69.63**	2.26
	REM	6.94	26.63	4.44	**61.99**

Garmin Vivosmart 4					
PSG	Wake	**27.64**	46.35	11.86	14.15
	N1 or N2	4.39	**60.33**	22.52	12.76
	N3	2.88	46.00	**47.46**	3.66
	REM	2.59	41.57	22.74	**33.10**

Apple Watch Series 8					
PSG	Wake	**52.15**	42.10	1.01	4.73
	N1 or N2	5.73	**83.27**	4.25	6.74
	N3	0.27	47.73	**50.66**	1.34
	REM	1.62	29.51	0.30	**68.57**

Each row in the error matrix is the sleep stage annotated by PSG, while each column represents the sleep stage annotated by the wearable. Values in bold indicate agreement in classification of sleep staging between PSG and wearable. Abbreviations: PSG, polysomnography; REM, rapid eye movement.

### Wrist positions

Overall, the kappa values of the different positions of the wrist were similar, ranging from 0.33 to 0.38, indicating moderate agreement between wearable measurements and the reference standard, regardless of wrist position. Sensitivity shows slight variation, with values such as 92.6% (left, lower position) and 95% (right, upper position). This suggests that certain positions may slightly enhance the detection of sleep/wake episodes. Specificity fluctuates, indicating differences in the accuracy of detecting wake states between positions.

The analysis showed that the performance of the wearables was quite similar across these wrist positions, with only minor variations observed in certain parameters such as TST and WASO. These variations were not substantial enough to indicate a meaningful impact of wrist position on overall performance. Thus, the findings suggest that the wearables performed consistently regardless of wrist placement (Supplementary Table S1).

## Discussion

This study assessed how well six different consumer wearable sleep-tracking devices detected sleep parameters compared to the gold standard, PSG.

All the wearable sleep-tracking devices, except the Fitbit Sense and the Fitbit Charge 5, significantly overestimated TST compared to PSG. For TST, wearable-based estimates are typically considered to be clinically satisfactory if the bias is less than 30 minutes [16]. Hence, even though the Apple Watch Series 8 displayed a significant overestimation of 19.60 minutes (*p* = 0.004) compared to PSG, the Apple Watch Series 8, as well as the Fitbit Charge 5 and Fitbit Sense, would be considered as clinically satisfactory for TST. The findings for WASO, SE, and SOL are consistent with many previous studies, which have similarly shown that wearables tend to overestimate SE and underestimate wake [5, 16, 28, 34–37]. In line with these studies, our results indicate that all the wearables significantly overestimated SE by 2.20% (*p* = 0.024) to 10.19% (*p* < 0.001), and significantly underestimated W (−11.76 to −39.57 minutes) and WASO (−12.38 to −47.94 minutes).

Although SE overestimations within a range of around 5% may be clinically acceptable for general wellness purposes, overestimations exceeding 10% could lead to significant discrepancies [38].

In addition, minor inaccuracies for WASO may be tolerable for healthy individuals. However, underestimations over 30 minutes are generally not considered acceptable for clinical or research-grade accuracy, as they could misinform about sleep fragmentation and overall sleep quality [39].

All wearable devices showed a sensitivity of >90% (ie, correct detection of sleep epochs). The Fitbit Sense (48.80%), Fitbit Charge 5 (47.51%), and the Apple Watch Series 8 (52.15%) showed higher specificity (ie, detecting wake epochs) compared to the Whoop 4.0 (40.13%), Withings Scanwatch (31.09%), and Garmin Vivosmart 4 (29.39%). Low specificity is a common finding when validating devices that rely predominantly on actigraphy to estimate sleep [25, 40–42].

Our findings in this study indicate that most wearable sleep-tracking devices significantly underestimate WASO and W, and significantly under- or overestimate LS compared to PSG during the same night. The detection of wake within sleep is difficult due to the similarity in movement between restful wake and sleep. Therefore, it is reasonable to suggest that the devices that are better at detecting wake within sleep, such as the Fitbit Sense and the Fitbit Charge 5, have refined their proprietary algorithms to improve wake detections. In addition, all the wearables use actigraphy and heart rate data which do not detect brief arousals or periods of LS as accurately as PSG’s monitoring of brainwaves and physiological signals. Our four-state categorization matrices also indicate that the main sources of error appear as misclassified PSG W, N3 sleep, and REM sleep as LS by the wearables. It is important to contextualize that LS makes up a significant portion of our total sleep time. On average, LS accounts for about 50%–60% of a typical night’s sleep. During LS, people are less active but still exhibit some movement. In addition, in LS, heart rate and breathing slow down but at a lesser extent as in DS or REM sleep. Since LS occupies a middle ground in terms of both heart rate and movement, it can become a “default” classification for wearables when there is uncertainty. Conservative algorithms are more likely to tend toward LS rather than overestimate critical stages like DS or REM when in doubt [8–10, 43–45]. Accordingly, all of our selected wearables tend to lean towards LS rather than DS or REM when classification is uncertain. Especially the Garmin Vivosmart 4 exhibits a pronounced tendency to misclassify multiple sleep stages as LS. With 46.35% of PSG W and 46.00% of PSG N3 epochs classified as LS, the Garmin Vivosmart 4 shows a strong tendency to treat LS as a fallback classification when in doubt.

Similar to previous research, our results showed differences for the measurements of DS and REM sleep [3, 16, 35]. All the wearable sleep-tracking devices, except the Fitbit Sense and Fitbit Charge 5, indicated significant over- or underestimations for DS. For REM sleep, the Whoop 4.0 and the Apple Watch Series 8 displayed a significant over- and underestimation compared to PSG, respectively. The Fitbit Charge 5, Fitbit Sense, and Garmin Vivosmart 4 showed no significant difference for measuring REM sleep compared to PSG. DS and REM sleep are generally measured more accurately by wearables due to their distinct physiological markers such as reduced heart rate and lack of physical movement compared to W and LS. Nevertheless, even measurements of DS and REM sleep obtained through wearables are not without limitations and can exhibit inaccuracies relative to PSG due to, for example, movement artifacts, algorithmic limitations, or external factors [44, 46]. Accordingly, our results showed that all wearables demonstrate a higher percentage of correctly identified epochs for DS and REM sleep compared to W and LS.

The variability in MAE and MAPE highlights that not all wearables are equally reliable for all sleep parameters. Devices like the Apple Watch Series 8 and Fitbit Charge 5 show more consistent accuracy, particularly for sleep parameters like TST and SE, making them more suitable for general sleep tracking. In contrast, other devices, such as the Garmin Vivosmart 4 and Withings Scanwatch, exhibit significant errors in specific areas (eg, WASO and DS), limiting their applicability for precise sleep analysis. Moreover, high errors in SOL and certain sleep stages suggest that wearables still face challenges in accurately identifying transitions and specific physiological states during sleep.

The analysis of the MAE and MAPE for sleep stage detection highlights considerable variability in performance among the wearables. Fitbit devices consistently exhibit moderate to high reliability, particularly in LS and REM detection. However, certain models, such as the Garmin Vivosmart 4, show substantial inaccuracies, particularly in W and DS measurements, as evidenced by their high MAPE values. These differences could be due to variations in sensor technology (eg, PPG, accelerometers) or algorithmic approaches.

The Cohen’s kappa coefficients of the wearable devices included in this study ranged from 0.21 to 0.53 when compared to PSG. The Cohen’s kappa values of the Fitbit Sense (0.42), Fitbit Charge 5 (κ = 0.41), and the Apple Watch Series 8 (κ = 0.53) indicate a moderate agreement between the device and PSG. The Cohen’s kappa values of the Whoop 4.0 (κ = 0.37), the Withings Scanwatch (κ = 0.22), and the Garmin Vivosmart 4 (κ = 0.21) indicate a fair agreement between the device and PSG.

However, it is crucial to contextualize the comparison of the wearables’ agreement with that of PSG, taking into consideration that the scoring of PSG is subject to variability among technicians [47]. As reported by Danker-Hopfe et al. [29], the interrater reliability is substantial rather than almost perfect (κ = 0.75). Given this benchmark, the devices with higher Cohen’s kappa coefficients such as the Fitbits and Apple Watch Series 8 appear to provide reasonable estimates of multistate sleep but need improvement to reach trained technician levels of agreement. However, it is reasonable to suggest that the devices which perform better at estimating multistate sleep could offer valuable insights for monitoring long-term changes in sleep stages. Therefore, a practical takeaway from this study is that devices with higher relative agreement for multistate sleep, such as the Fitbit Sense, Fitbit Charge 5, and Apple Watch Series 8, can be used to track prolonged and significant changes in sleep architecture.

While wearables do not replace PSG for clinical diagnosis, they offer a practical solution for longitudinal sleep monitoring outside the laboratory, which can provide valuable supplementary data for healthcare professionals. In research, reliable sleep tracking enables large-scale epidemiological studies on sleep and its impact on health, reducing reliance on costly and time-intensive PSG recordings. For personal use, wearables can help individuals gain insights into their sleep patterns, identify trends, and implement behavioral changes to improve sleep hygiene. However, the clinical utility of these devices depends on their accuracy in detecting sleep stages, as misclassifications may lead to misleading interpretations of sleep quality. Therefore, while some devices, such as the Fitbit Charge 5, Fitbit Sense, and Apple Watch Series 8, demonstrate clinically acceptable accuracy for certain parameters, users should remain cautious about relying on these devices for detailed sleep staging without validation against PSG.

While this study demonstrates that, for example, some wearable devices demonstrate clinically acceptable accuracy in measuring TST and SE and can accurately measure sleep stages such as DS and REM sleep, it is important to emphasize that these devices currently do not serve as replacements for PSG in clinical diagnoses since precise sleep staging is crucial for diagnosing and monitoring sleep disorders such as insomnia, sleep apnea, and narcolepsy. PSG remains the gold standard for sleep assessment, offering comprehensive data and analysis crucial for diagnosing sleep disorders. In research, reliable sleep tracking enables large-scale studies on sleep and its impact on health, reducing reliance on costly and time-intensive PSG recordings. For personal use, wearables can provide valuable insights into an individual’s sleep architecture over time and could help identify subtle changes in sleep quality or architecture which could be supplementary data for healthcare professionals. However, it is still critical for users of wearable devices to be aware of the inherent margin of error and that the results may deviate from actual sleep states [17–19].

### Limitations

A primary limitation of this study is that while participants wore the wearable sleep-tracking devices from late afternoon until the following morning (covering the period before, during, and after the PSG session), validating the performance of sleep parameters was restricted to the lights-out period corresponding to the PSG recording. Consequently, the limited sleep data captured by the wearable outside of the PSG-validated period could not be validated against PSG and may affect the generalizability of the results to real-world sleep conditions. Secondly, future research would benefit from conducting wearable performance validation over multiple nights, as this would provide a more comprehensive understanding of the wearables’ performance across various nights and sleep conditions. While wearables are designed to provide immediate insights, wearing them over multiple nights could improve data accuracy by allowing the devices to adapt to individual baseline patterns and to account for missing or (partial) data loss. Furthermore, using proprietary algorithms for sleep estimation in wearable devices could diminish the consistency over time and across studies, since these algorithms are periodically updated. Longitudinal comparisons of sleep architecture may be affected, limiting the ability to assess long-term trends and changes reliably. Additionally, variations in algorithms between different devices can hinder cross-study comparisons, making it challenging to generalize findings or establish standardized benchmarks. Future research should address these challenges by advocating for greater transparency in algorithmic updates and exploring methods to standardize sleep measurement across devices.

A third limitation is the slight variation in data points across wearables, especially for the Garmin Vivosmart 4 and Apple Watch Series 8. The variation in data points is primarily due to the delayed acquisition of the Apple Watch Series 8 because of logistical restraints, and missing data or (partial) data loss. While these variations may introduce minor inconsistencies in direct comparisons, the overall trends and insights remain robust. Ensuring more balanced amount of datasets across devices in future research could enhance the precision of comparative analyses and strengthen the generalizability of findings. Lastly, regarding the participant sample, future studies should enhance the age range by including both younger and older individuals to capture a broader range of sleep architectures. Additionally, the study sample consisted of 52 males and 10 females, resulting in a gender imbalance. This distribution reflects the demographic characteristics of the patient population attending the sleep center during the recruitment period, which comprises a higher proportion of males. Efforts were made to recruit female participants as well. However, the availability of eligible female subjects was limited. Furthermore, detailed data on participants’ specific skin tones and racial/ethnic backgrounds were not systematically collected. This is particularly relevant for technologies utilizing PPG, as skin tone can influence PPG accuracy and, consequently, the sleep estimates derived from wearable algorithms. While most participants were Caucasian and had lighter skin tones, the results can not be fully generalized to individuals with darker skin tones. Future studies should aim to recruit a more balanced sample to account for the variability in sleep architecture during the life span and to explore potential gender-specific differences in wearable device performance. In addition, skin tone and race/ethnicity should be systematically collected to better understand the impact of these factors on wearable device performance.

This study included participants with as well as without sleep apnea, allowing for a larger sample size. After analyzing the performance of the wearable between groups with different sleep apnea severity, it was seen that the agreement between the wearables and PSG tend to decrease as sleep apnea severity increases. However, due to the unequal sample sizes between these groups (no sleep apnea: *n* = 27, mild sleep apnea: *n* = 18, moderate sleep apnea: *n* = 12, severe sleep apnea: *n* = 3, extremely severe sleep apnea: *n* = 2), definite conclusions regarding wearable performance in individuals with varying apnea severity could not be drawn. Future studies with more balanced sample sizes for sleep apnea severity are mandatory to confirm and clarify these findings (Supplementary Table S2).

Lastly, the three-state classification system employed by the Withings Scanwatch, which consolidates REM sleep and DS into a single “deep sleep” category, differs significantly from the four-state categorization used by the other wearables. This methodological discrepancy can have an impact on the findings when evaluating the performance of wearables. The aggregation of REM and DS by the Withings device may limit the comparability of its performance with other wearables that adhere to the four-state model.

## Conclusion

This study highlights the varying accuracy of six consumer wearable devices in detecting sleep parameters compared to the gold standard, PSG. While some wearables, such as the Fitbit Sense, Fitbit Charge 5, and Apple Watch Series 8, demonstrate clinically acceptable accuracy in measuring TST and SE, discrepancies remain across other sleep metrics, including WASO, and the accurate differentiation of LS and W epochs. However, the higher Cohen’s kappa coefficients of the wearable devices, such as the Fitbit Sense, Fitbit Charge 5, and Apple Watch Series 8, suggest that these wearables could serve as effective tools for tracking general trends and long-term changes in sleep architecture

While wearables do not match PSG in clinical accuracy and should not replace it for diagnostic purposes, their accessibility, ease of use, and potential for real-time monitoring make them valuable for observing sleep patterns outside the sleep clinic. As wearables continue to evolve, these devices may offer increasingly valuable insights into sleep architecture and quality, especially when used to complement PSG assessments or track individual sleep trends over time. However, users must remain aware of the inherent inaccuracies in wearable data, as these devices do not replace PSG for clinical diagnoses. Future improvements in the wearable algorithms may further bridge the gap with PSG.

In addition, future research should focus on validating the performance of newer models and exploring the long-term implications of wearable data, aiming to enhance the reliability of these tools for tracking sleep health and their potential utility in (pre-)clinical monitoring settings.

## Supplementary Material

zpaf021_suppl_Supplementary_Materials

## Data Availability

The data underlying this article will be shared on reasonable request to the corresponding author.
