# Supplementary material for: A performance validation of six commercial wrist-worn wearable sleep-tracking devices for sleep stage scoring compared to polysomnography
Source: Sleep Adv. 2025 Mar 22;6(2):zpaf021. doi: 10.1093/sleepadvances/zpaf021 (PMC12038347; doi:10.1093/sleepadvances/zpaf021)
Supplement: zpaf021_suppl_Supplementary_Materials [file zpaf021_suppl_supplementary_materials.docx]

# **A performance validation of six commercial wrist-worn wearable sleep-tracking devices for sleep stage scoring compared to polysomnography**

An-Marie Schyvens^1,2^; Brent Peters^4^; Nina Catharina Van Oost^3^; Jean-Marie Aerts^3^; Federica Masci^3^; An Neven^4^; Hélène Dirix^4^; Geert Wets^4^; Veerle Ross^4,5^; Johan Verbraecken^1,2^

1 Laboratory of Experimental Medicine and Pediatrics, University of Antwerp, Wilrijk, Belgium

2 Multidisciplinary Sleep Disorders Centre, Antwerp University Hospital, Edegem, Belgium

3 Department of Biosystems, KU Leuven, Leuven, Belgium

4 UHasselt – Hasselt University, Transportation Research Institute (IMOB), Martelarenlaan 42, Hasselt 3500, Belgium

5 Faresa, Evidence-Based Psychological Centre, Hasselt, Belgium

Corresponding Author:

An-Marie Schyvens, MSc

Multidisciplinary Sleep Disorders Centre

Antwerp University Hospital

Drie Eikenstraat 655

Edegem, 2650

Belgium

Phone: 32 497 62 71 00

Email: [an-marie.schyvens@uantwerpen.be](mailto:an-marie.schyvens@uantwerpen.be)

| Table S1. Differences in performance per wrist position displayed as the biases between the wearables and polysomnography (PSG). | | | | | | | | | | | | |
| --- | --- | --- | --- | --- | --- | --- | --- | --- | --- | --- | --- | --- |
| **Wrist Position** | **n** | **kappa** | **Sensitivity (%)** | **Specificity (%)** | **TST (min)** | **WASO (min)** | **SE (%)** | **SOL (min)** | **W (min)** | **LS (min)** | **DS (min)** | **REM (min)** |
| Left, lower | 55 | 0.38 | 92.6 | 42.1 | 15.2 | -23.8 | 5.7 | -0.8 | -48.9 | 13.6 | 6.4 | 3.2 |
| Left, upper | 40 | 0.33 | 94.4 | 40.0 | 28.5 | -30.7 | 6.5 | 0.8 | -58.6 | -20.5 | 31.5 | 18.3 |
| Right, lower | 49 | 0.34 | 91.4 | 45.7 | 10.5 | -24.8 | 5.2 | 14.1 | -27.5 | -8.9 | 35.9 | -14.5 |
| Right, upper | 44 | 0.34 | 95.0 | 36.1 | 36.3 | -29.7 | 6.3 | -8.7 | -69.1 | 10.8 | 22.7 | 1.1 |

| Table S2. The performance of the wearables between groups with different sleep apnea severity. | | | | | |
| --- | --- | --- | --- | --- | --- |
| **Apnea Severity (AHI)** | **Wearable** | **n** | **Kappa** | **Sensitivity (%)** | **Specificity (%)** |
| **No apnea (<5/h)** | Fitbit Sense | 18 | 0.42 | 92.40 | 47.89 |
|  | Fitbit Charge 5 | 16 | 0.41 | 91.78 | 49.11 |
|  | Whoop 4.0 | 19 | 0.38 | 94.97 | 38.39 |
|  | Withings Scanwatch | 18 | 0.21 | 94.79 | 29.75 |
|  | Garmin  Vivosmart 4 | 13 | 0.18 | 96.08 | 26.78 |
|  | Apple Watch Series 8 | 8 | 0.47 | 97.00 | 44.83 |
| **Mild (5-15/h)** | Fitbit Sense | 10 | 0.45 | 95.20 | 47.97 |
|  | Fitbit Charge 5 | 11 | 0.45 | 92.51 | 51.52 |
|  | Whoop 4.0 | 10 | 0.37 | 88.98 | 47.04 |
|  | Withings Scanwatch | 10 | 0.28 | 97.86 | 42.05 |
|  | Garmin  Vivosmart 4 | 7 | 0.21 | 93.96 | 33.86 |
|  | Apple Watch Series 8 | 5 | 0.53 | 96.71 | 58.12 |
| **Moderate (15-30/h)** | Fitbit Sense | 7 | 0.39 | 94.44 | 52.86 |
|  | Fitbit Charge 5 | 7 | 0.36 | 90.66 | 49.67 |
|  | Whoop 4.0 | 4 | 0.30 | 95.24 | 48.61 |
|  | Withings Scanwatch | 8 | 0.19 | 88.09 | 34.16 |
|  | Garmin  Vivosmart 4 | 3 | 0.18 | 99.88 | 20.52 |
|  | Apple Watch Series 8 | 3 | 0.56 | 97.67 | 58.71 |
| **Severe (30-50/h)** | Fitbit Sense | 1 | 0.49 | 86.27 | 61.24 |
|  | Fitbit Charge 5 | 2 | 0.42 | 90.62 | 38.68 |
|  | Whoop 4.0 | 2 | 0.21 | 92.57 | 30.89 |
|  | Withings Scanwatch | 2 | 0.27 | 99.59 | 9.83 |
|  | Garmin  Vivosmart 4 | 1 | 0.06 | 100.00 | 2.81 |
|  | Apple Watch Series 8 | 0 | N/A | N/A | N/A |
| **Extremely severe (50-80/h)** | Fitbit Sense | 0 | N/A | N/A | N/A |
|  | Fitbit Charge 5 | 2 | 0.42 | 95.71 | 33.15 |
|  | Whoop 4.0 | 2 | 0.34 | 97.87 | 11.27 |
|  | Withings Scanwatch | 0 | N/A | N/A | N/A |
|  | Garmin  Vivosmart 4 | 0 | N/A | N/A | N/A |
|  | Apple Watch Series 8 | 1 | 0.46 | 87.40 | 60.99 |
| **N/A: not applicable.** | | | | | |
